# Recent Progress in Biosensors Based on Biorecognition Molecules

**DOI:** 10.3390/bios13090842

**Published:** 2023-08-24

**Authors:** Zhen Zhang

**Affiliations:** School of the Environment and Safety Engineering, Jiangsu University, Zhenjiang 212013, China; zhangzhen@ujs.edu.cn

Biosensors are considered a popular technology to rapidly detect targets, and are generally composed of biorecognition molecules that specifically capture analytes and signal elements. In terms of methodology, measurements are carried out in a limited time without sophisticated sample pre-treatments, implying obvious advantages over traditional instrument methods for rapid analysis [1].

Considering that biosensors are widely applied in environmental pollutant monitoring, detecting hazardous substances in foods and disease diagnosis [2,3,4], their advances and future trends should be highlighted, so we have organized this Special Issue entitled “Bioassays and Biosensors for Rapid Detection and Analysis”. Among the 10 papers published, 6 are articles, 3 are communications and 1 is a review.

Huang et al. believe that electrogenerated chemiluminescence (ECL) is a powerful tool for sensitive and accurate detection of biological analytes and summarize the recent advances in this field. Moreover, some future trends and challenges of ECL biosensors are also discussed. They believe that after integration with low-cost photodetectors, ECL biosensors will have great potential in commercial applications [5]. Meanwhile, several works focus on contaminant determination in food and environments, like sulfadiazine (SDZ) [6], deoxynivalenol (DON) [7] and dibutyl phthalate (DBP) [8]. With regard to the detection systems, antibodies or aptamers were used to recognize the targets, which contributed to good selectivity. In addition, some novel strategies for signal amplification were employed to improve their analytical performance [8]. At the same time, sensitive biosensors were fabricated for the rapid detection of the biomarkers related to some diseases, which could be achieved easily [9,10].

Most studies focus on the sensitivity improvement of biosensors through introducing new functional materials or simplifying the measurement procedure, which are definitely important. However, more attention should be paid to real applications, increasing tolerances against various matrices that potentially influence the performance of biosensors. Interestingly, in this Special Issue, two papers are related to commercial products using bioassays or biosensors [11,12]. In short, biosensors will be widely used in various fields after some problems have been overcome, including achieving satisfactory sensitivity and accuracy and maintaining enzyme activity in varied harsh environments.
